# The challenging journey of cervical cancer diagnosis and treatment at the second largest hospital in Indonesia

**DOI:** 10.1016/j.gore.2024.101325

**Published:** 2024-01-18

**Authors:** Brahmana Askandar Tjokroprawiro, Khoirunnisa Novitasari, Wita Saraswati, Indra Yuliati, Renata Alya Ulhaq, Hanif Ardiansyah Sulistya

**Affiliations:** Departement of Obstetrics and Gynecology, Faculty of Medicine, Universitas Airlangga, Dr. Soetomo General Academic Hospital, Jl. Prof. Dr. Moestopo 6-8, Surabaya 60286, Indonesia

**Keywords:** Cervical cancer, Screening, Treatment

## Abstract

**Objectives:**

Cervical cancer is a major health concern in developing countries. Access to preventive measures is limited in low- and middle-income countries, and cervical cancer is often identified at an advanced stage of the disease. In this study, we aimed to investigate when patients were first diagnosed and received treatment at a large hospital in Indonesia.

**Methods:**

Data were collected using a questionnaire from outpatient visits and descriptively analyzed. Totally, 215 cervical cancer patients being treated at the Dr. Soetomo Academic Hospital's gynecological oncology outpatient clinic in Indonesia between August and October 2022 were included.

**Results:**

Most patients were 51–60 years old (36.3 %), housewives (87 %), and had an elementary school education level (50 %). Most (88.4 %) admitted they were unaware about cervical cancer prevention, and 85.6 % never underwent screening. Most cervical biopsies were performed at primary hospitals (42.3 %). Fear of cancer treatment was the most frequent reason for patients arriving late at tertiary hospitals (50 %). Treatment delays occurred because patients had to visit two healthcare facilities before visiting a tertiary hospital (47.4 %). Most patients were diagnosed with stage III cancer (38.1 %), and chemotherapy was administered as the first-line therapy (96.3 %). Most patients (51.2 %) received their first therapy >12 months after initial symptom onset.

**Conclusions:**

Most cervical cancer patients were diagnosed at a late stage owing to a lack of information regarding early symptoms and irregular screenings. Treatment was delayed owing to social barriers. Therefore, the government should have more strict policies to implement cervical cancer detection and prevention.

## Highlights

• Most patients with cervical cancer did not know about preventive measures and never underwent screening.
• Most patients were diagnosed at a late stage due to lack of knowledge regarding early symptoms and irregular screenings.
• Most patients received their first treatment >1 year after symptom onset.
• Delays in visiting tertiary hospitals for treatment were mostly due to fear of cancer therapy.
• Other reasons for treatment delays were complicated referral pathways and exploring alternative medicines.

## 1 Introduction

Globally, cervical cancer is the fourth most common cancer in women, with an estimated 604,000 new cases and 324,000 deaths in 2020. Approximately 90 % of these new cases and deaths occurred in low- and middle-income countries (Sung et al., 2021). Women living with human immunodeficiency virus (HIV) are six times more likely to develop cervical cancer compared with women without HIV; HIV causes an estimated 5 % of all cervical cancer cases (Stelzle et al., 2021).

In high-income countries, targeted programs allow children to receive the human papillomavirus (HPV) vaccination and women to be regularly screened and adequately treated. However, access to these preventive measures is limited in low- and middle-income countries, and cervical cancer is often not identified until the disease has advanced and symptoms have developed. In addition, access to treatments for cancerous lesions (such as cancer surgery, radiotherapy, and chemotherapy) may be limited, resulting in higher mortality rates from cervical cancer in these countries.

In Indonesia, cervical cancer remains the most common gynecological cancer with the second largest number of patients after breast cancer. According to GLOBOCAN 2020 data, the incidence of cervical cancer in Indonesian women is approximately 36,633 cases (9.2 % of new cancer cases in both sexes and all ages), with a mortality rate of 21,003 (9 %) (The Global Cancer Observatory, 2020).

Current early detection methods for cervical cancer include visual inspection with acetic acid, liquid-based pap test, and HPV DNA testing. Human papillomavirus DNA testing is performed to detect early infection by high-risk HPV that can cause cervical cancer. In Indonesia, HPV subtypes 16, 18, 45, and 52 are common high-risk subtypes (Murdiyarso et al., 2016). Cervical cancer generally does not exhibit symptoms at an early stage. The presence of symptoms usually indicates that the disease has reached the pre-cancerous or even cancerous stage. Therefore, early detection through routine screening is imperative.

Awareness of cervical cancer screening and prevention is lacking among women and health workers, especially in developing countries (Dulla et al., 2017). A *meta*-analysis showed that theory-based cervical cancer education is required to raise awareness and increase women’s participation in cervical cancer screening programs (Musa et al., 2016). Reluctance to undergo screening can result in delays in diagnosis and treatment, which is problematic for healthcare systems worldwide. A systematic review demonstrated that a four-week delay in cancer treatment is associated with an increased mortality rate across all surgical, systemic treatment, and radiotherapy indications for seven types of cancer (Hanna et al., 2020).

Policies that minimize system-level delays in cancer treatment initiation can improve population-level survival outcomes (Hanna et al., 2020). Therefore, we aimed to elucidate the timing of cervical cancer diagnosis and the initiation of treatment at Dr. Soetomo General Academic Hospital in Surabaya, Indonesia. Dr. Soetomo General Academic Hospital is a tertiary hospital in Indonesia’s second largest city, Surabaya, and serves as a referral hospital for 40 million people in East Java Province. The patient population at this hospital is very diverse in terms of regional origin, ethnicity, religion, and social status.

## 2 Materials and methods

### 2.1 Patient population & data gathering

This retrospective observational study included 215 women diagnosed with cervical cancer who were referred to Dr. Soetomo General Academic Hospital in Surabaya, Indonesia, and registered at the gynecological oncology outpatient clinic between August and October 2022. Patients diagnosed with cervical cancer undergoing treatment at Dr. Soetomo General Academic Hospital who had received at least one therapy were included in this study. Data were obtained via direct interviews using a questionnaire.

### 2.2 Data & statistical analysis

The questionnaire assessed the characteristics, screening, and therapy of patients with cervical cancer, as well as the journey from their first consultation to their first treatment at Dr. Soetomo General Academic Hospital Surabaya. The results were analyzed and presented in descriptive form.

The study protocol was approved by the Institutional Review Board of Dr. Soetomo General Academic Hospital (reference number: 0488/KEPK/IX/2022). All patients provided written informed consent before participating in this study.

## 3 Results

### 3.1 Sociodemographics

**Fig. 1:**
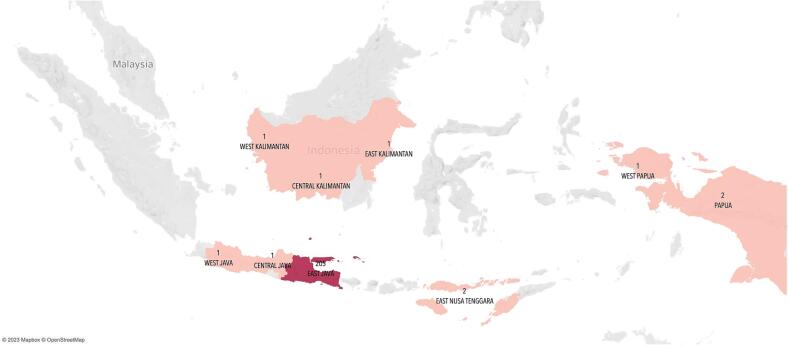
Distribution of patients by region.

**Fig. 2:**
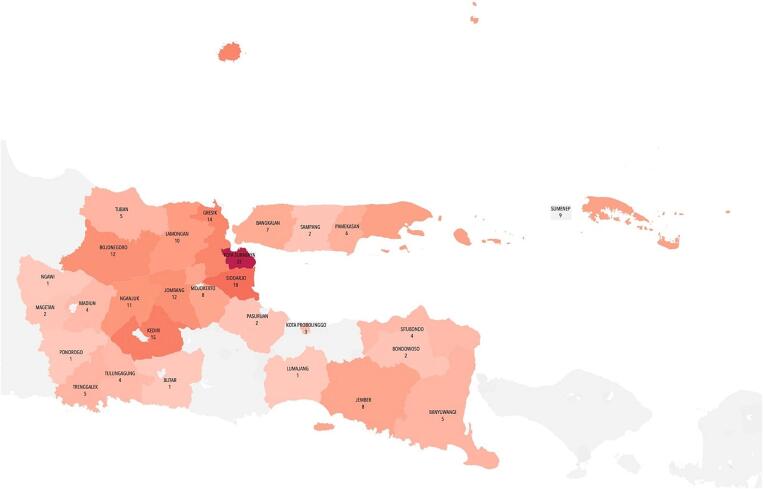
Distribution of patients by city/district in East Java.

Most of the 215 patients included in this study were aged 51–60 years (36.28 %) and resided in East Java Province, with only 10 (4.6 %) residing elsewhere (Figs. 1 and 2). A majority of the patients were housewives (86.97 %) and had an elementary school education level (49.99 %) (Table 1).

**Table 1:** Participant characteristics.

Characteristics	N (%)
*Age (years)*	
21–30	4 (1.86)
31–40	29 (13.48)
41–50	58 (26.97)
51–60	78 (36.28)
61–70	40 (18.60)
>70	6 (2.79)

*Region*	
East Java	205 (93.35)
Outside East Java	10 (4.65)

*East Java Area*	
Surabaya	33 (16.09)
Outside Surabaya	172 (83.90)

*Profession*	
Housewife	187 (86.97)
Entrepreneur	13 (6.05)
Government employee	3 (1.39)
Private sector employee	5 (2.33)
Other	7 (3.26)

*Highest Education Level*	
No school	2 (0.93)
Elementary school	107 (49.99)
Junior high school	44 (20.47)
Senior high school	45 (20.93)
Bachelor’s degree	16 (7.44)
Master’s degree	1 (0.47)

### 3.2 Screening & prevention

The patient symptoms described in Table 2 show that abnormal uterine bleeding, chronic vaginal discharge, and pelvic pain were the three most common symptoms of cervical cancer. Most patients visited a healthcare provider >12 months after experiencing their first symptoms. In total, 190 (88.37 %) patients did not know about cervical cancer prevention and 85.58 % had never undergone screening. The most frequently used screening method was a pap test, which was performed in hospitals, mostly by obstetricians. A public health center was the most visited healthcare facility after patients experienced their first symptoms.

**Table 2:** Data on cervical cancer screening and prevention.

Screening and Prevention	N (%)
*Chief complaint leading to consultation with a HCP*	
Abnormal uterine bleeding	168 (29.31)
Post-coital bleeding	81 (14.14)
Chronic vaginal discharge	140 (24.43)
Pelvic pain	99 (17.28)
Increased frequency of urination	39 (6.81)
Defecation disorders	39 (6.81)
Fatigue	7 (1.22)

*Interval between first symptoms and first visit to healthcare facility*	
1–3 months	36 (16.74)
4–6 months	50 (23.26)
7–9 months	11 (5.12)
9–12 months	17 (7.91)
>12 months	101 (46.97)

*Knowledge about cervical cancer*	
Yes	25 (11.63)
No	190 (88.37)

*Routine screening*	
Never	184 (85.58)
Yes	4 (1.86)
No	27 (12.56)

*Reason*	
No symptoms	24 (88.89)
Scared/uncomfortable	3 (11.11)

*Last screening*	
1–2 years ago	12 (38.71)
3–5 years ago	9 (29.03)
>5 years ago	10 (32.26)

*Screening method*	
VIA	7 (22.58)
Pap smear	24 (77.42)

*Screening location*	
Public health center	8 (25.8)
Hospital	19 (61.29)
Private laboratory	1 (3.23)
Private clinic	3 (9.67)

*Screening provider*	
Midwife	9 (29.03)
General practitioner	3 (9.67)
Obstetrician	18 (58.06)
Laboratory	1 (3.23)

*Health provider visited after first symptoms*	
Midwife	59 (27.4)
General practitioner	49 (22.79)
Obstetrician	107 (49.77)

*Healthcare facility visited after the first symptoms*	
Private midwife clinic	40 (18.6)
Private general practitioner clinic	15 (6.98)
Private obstetrician clinic	37 (17.21)
Public health center	51 (23.72)
Primary hospital	29 (13.49)
Secondary hospital	42 (19.53)
Tertiary hospital	1 (0.47)
Private laboratory	0 (0)

HCP, Healthcare provider; VIA, Visual Inspection with Acetic acid.

A pap test also could be performed in a primary health care center; however, there are only 139 pathologists in East Java with 64 of them in Surabaya. The Indonesian Cancer Foundation also supports cervical cancer patients. However, they cannot reach all the patients, as currently, the Indonesian Cancer Foundation is not available in every city in Indonesia. Our limited resources and funding may result in limited coverage.

### 3.3 Treatment characteristics

Table 3 describes patient diagnoses and therapy pathways. The histopathological diagnosis of cervical cancer was mainly performed by cervical biopsy in primary hospitals (42.33 %). Approximately 26 patients (12.1 %) delayed their referral visit at tertiary hospitals by one month from being histopathologically diagnosed (46.31 %), mostly because they were afraid of cancer treatment (50 %), tried to seek alternative medicine (23.08 %), or had financial problems (11.54 %).

**Table 3:** Cervical cancer diagnosis- and treatment-related information.

Diagnosis- and Treatment-related Information	N (%)
*Place of diagnosis*	
Private midwife clinic	0 (0)
Private general practitioner clinic	0 (0)
Private obstetrician clinic	16 (7.44)
Public health center	1 (0.47)
Primary hospital	91 (42.33)
Secondary hospital	79 (36.74)
Tertiary hospital	25 (11.63)
Private laboratory	0 (0)

Interval between first visit to healthcare facility and histopathological confirmation	\
1–3 months	133 (61.86)
4–6 months	41 (19.07)
7–9 months	2 (0.93)
9–12 months	29 (13.49)
>12 months	10 (4.65)

*Source of payment for histopathological diagnosis*	
Self-payment	46 (21.4)
Government insurance	168 (78.14)
Private insurance	1 (0.47)

*Patient delay to first tertiary hospital visit*	
Yes	26 (12.1)

*Time from histopathological diagnosis to first visit to tertiary hospital*	
1 month	11 (46.31)
2–3 months	4 (15.38)
4–6 months	4 (15.38)
7–12 months	6 (23.08)
>12 months	1 (3.85)

*Reason for delay*	
Cost	3 (11.54)
No companion/distance between home and referral hospital	2 (7.69)
Tried alternative medicine	6 (23.08)
COVID-19 pandemic	2 (7.69)
Afraid of cancer treatment	13 (50)
No	189 (87.9)

*Number of healthcare facilities visited before Dr. Soetomo General Hospital*	
1	61 (28.37)
2	102 (47.44)
3	44 (20.46)
4	6 (2.79)
5	2 (0.93)

*Knowledge of cancer stage*	
Stage I	4 (1.86)
Stage II	66 (30.7)
Stage III	82 (38.14)
Stage IV	5 (2.33)
Don’t know	58 (26.98)

*Interval between histopathological diagnosis and first treatment*	
<1 month	156 (72.56)
2–3 months	34 (15.81)
4–6 months	10 (4.65)
7–12 months	14 (6.51)
>12 months	1 (0.47)

*First treatment method*	
Radiation only	3 (1.4)
Chemotherapy only	207 (96.3)
Surgery	5 (2.33)

*Interval between first visit to tertiary hospital and first treatment at tertiary hospital*	
<1 month	14 (34.15)
2–3 months	8 (19.51)
4–6 months	7 (17.07)
7–12 months	11 (26.83)
>12 months	1 (2.44)

*Patient reason for delayed treatment at tertiary hospital*	
Cost	5 (9.36)
No companion/distance between home and referral hospital	8 (14.81)
Tried alternative medicine	9 (16.67)
COVID-19 pandemic	5 (9.36)
Afraid of cancer treatment	15 (27.78)
Complicated referral path	12 (22.22)

*Interval between first symptoms and initial treatment*	
<1 month	13 (6.05)
2–3 months	29 (13.49)
4–6 months	50 (23.26)
7–12 months	13 (6.05)
>12 months	110 (51.16)

COVID-19, coronavirus disease.

Indonesia established a national public insurance system in 2014 that covers the medication of patients including cancer treatment. However, it does address all challenges, since it only covers treatment costs and does not include daily expenses of their family and loss of pay while the patient is unable to work.

Before patients reached tertiary hospitals and received definitive therapy, most had visited two healthcare facilities (47.44 %); six patients (2.79 %) had visited four healthcare facilities. Most patients were diagnosed with stage III cancer (38.14 %) when they first visited our gynecological oncology outpatient clinic, and most received chemotherapy as their first therapy (96.3 %). In total, 41 patients (19.07 %) had a 4–6-month interval between their first visit to a healthcare facility and histopathological confirmation. Interval from the onset of symptoms to treatment exceeded 12 months in 52 % of the patients.

## 4 Discussion

### 4.1 Summary of main results

This study shows the journey of patients with cervical cancer from the first consultation until the initiation of treatment. It highlights the advanced stage of presentation and underlying social factors that contribute to poor outcomes. Most causes were related to patient problems. Most patients lacked awareness regarding cervical cancer, lacked access to screening and providers, and had visited multiple healthcare facilities before visiting the cancer center.

### 4.2 Results in the context of published literature

The patients with cervical cancer in our hospital were mainly 51–60 years old, and most were not professionally employed (homemakers). A cross-sectional study indicated that occupation is a protective factor against delayed diagnosis Ouasmani et al. (2017). In our patient cohort, the most common highest educational level was elementary school. These findings indicate that a low level of education—such as no education or elementary school—increases the risk of delayed cervical cancer diagnosis (Traoré et al., 2020). Most of our patients with cervical cancer had delays in seeking treatment, with an interval between their first symptoms and their first visit to a healthcare provider exceeding 90 days (Gyenwali et al., 2014, Ouasmani et al., 2017).

The Indonesian government already has some health promotion programs specifically targeting cervical cancer. However, they do not cover all of Indonesia as this is an archipelago country. Therefore, most people’s understanding about cervical cancer remains limited. Besides that, health information about cervical cancer is not taught in school. This is similar to the status in other Asian countries. A questionnaire study in Asia-Oceania shows that approximately 10–15 % did not have a national vaccination or screening program. The estimated coverage rate for vaccination and screening varied from below 1 % to 70 %. Notably, these coverage rates demonstrated a parallel association with the incidence and mortality rates of cervical cancer (Tse et al., 2023).

Most patients with cervical cancer had a delayed diagnosis. In this study, delayed diagnosis was defined as an interval of >30 days between the first visit to a healthcare provider and a histologically confirmed diagnosis (Gyenwali et al., 2013). The median time taken for a cervical cancer diagnosis based on histopathological results was 146 days. Diagnostic delay was associated with the level of the healthcare facility that was first contacted and the number of healthcare facilities visited before diagnosis.

The most common symptom that led patients to consult with a healthcare provider was abnormal uterine bleeding. This finding is in line with that of a previous cross-sectional study that identified abnormal vaginal bleeding as the most common symptom in patients with cervical cancer (Ouasmani et al., 2017). Our patients also experienced other symptoms, such as chronic vaginal discharge, pelvic pain, or post-coital bleeding, but many reported not recognizing this as a possible cancer symptom. A cross-sectional study by Lourenço et al. showed that vaginal discharge was more common among patients who experienced delays in visiting healthcare providers (Lourenço et al., 2012). Most of our patients were unaware of cervical cancer and had never undergone screening. Six previous studies stated that this lack of awareness of the symptoms of cervical cancer is a risk factor for delayed diagnosis. Most patients delay seeking medical care because they think the symptoms will resolve by themselves and will not be bothered by their first symptoms (Allahqoli et al., 2022). Our study shows that nearly half of patients delayed seeing a healthcare provider for >12 months after experiencing their first symptoms.

Most patients who underwent screening did not undergo routine screening because they experienced no specific symptoms or had no complaints and were afraid to be examined; this is in line with a previous study reporting that fear of cervical cancer diagnosis and not having a perception of severe disease increase the risk of delayed diagnosis (Allahqoli et al., 2022). Lack of awareness and the belief that cervical cancer is preventable may also be barriers to screening. Notably, 12/31 patients underwent their last screening 1 year before their cervical cancer diagnosis. This indicates that the screening method was inadequate. The most common screening method was a pap test because it was easy and affordable for the patient. Additionally, most of our patients underwent screening at a primary hospital performed by an obstetrician. However, first-line screening is supposed to be accessible, being performed by primary healthcare providers. Multiple barriers to screening were reported, including a dislike of pelvic examinations, embarrassment at being screened by a male doctor, anxiety over the cost, fearing a positive result, and being asymptomatic and not perceiving the need for screening [13].

Most patients visited obstetricians in private clinics or primary hospitals after experiencing their first symptoms. Unfortunately, they did not visit the healthcare provider directly after experiencing symptoms. Approximately half of the patients sought treatment 12 months after their first symptoms because they thought the symptoms would disappear. Biopsy of the cervical mass was primarily performed in primary and secondary hospitals. The interval between the first visit to a healthcare provider and histopathological confirmation was mostly 1–3 months. Most patients underwent cervical biopsy for diagnosis under government insurance; therefore, they had to follow a referral pathway from public healthcare services to primary hospitals. After being diagnosed with cervical cancer, 12.1 % of the patients delayed referral to cancer centers at tertiary hospitals. Fear of cancer therapy and exploring alternative treatments emerged as common reasons. Most patients visited two healthcare facilities before reaching the tertiary cancer center, and 3.72 % of our patients visited four to five healthcare providers before visiting our cancer center. Diagnostic delays were significantly associated with the level of the healthcare facility that was first contacted and the number of different healthcare facilities visited before diagnosis (Dereje et al., 2020). The delay was three times higher in patients who visited more than four other healthcare facilities for a cancer diagnosis (Dereje et al., 2020).

The incidence of delayed treatment was relatively low in our patients (Gyenwali et al., 2014, Ouasmani et al., 2017). Most of our patients with cervical cancer were administered initial therapy within 1 month after the first diagnosis. Some patients delayed therapy after visiting a tertiary hospital for social reasons. Most were afraid of cancer treatment, had complicated referral pathways, and wanted to explore alternative medicines. In Indonesia, social environments including friends and families are influential in guiding patients to seek treatment. In our social culture, the patient likely experienced social discrimination because they were labeled as a suffering person that has low survival and adds more burden to their society (Spagnoletti et al., 2020). The side effects of cancer treatment such as nausea, hair loss, and pain are also worrying for the patients.

The most common interval between symptom onset and the initiation of therapy in our patient population was >12 months. Inadequate patient knowledge and insufficient patient compliance may be at the root of this problem. Most of our patients visited private or primary healthcare providers when they experienced their first symptoms because it was the nearest place to seek consultation.

However, women in Nepal who initially visited a government hospital had a lower risk of delayed cervical cancer diagnosis than those referred to other healthcare facilities (Gyenwali et al., 2013). This is because healthcare providers working at the primary care level are more likely to be nurses or health officers who are less likely to be knowledgeable about cervical cancer for prompt referral of patients with relevant symptoms. Furthermore, the availability of pathologists for histological diagnosis is a relevant concern. In the US, there are 57 pathologists per 1 million people (Robboy et al., 2013), while in Indonesia, there are three pathologists per 1 million people, which is far from ideal.

In terms of the high incidence of cervical cancer, the major problems are the low levels of cervical cancer screening and limited resources of healthcare providers in remote areas in Indonesia. Besides these factors, the biggest issues that play a role in delayed cancer diagnosis comprise social matters, including lack of knowledge and cancer awareness, a low level of perceived risk, minimal signs and symptoms in the initial stage, personal beliefs, fear of cancer treatment, cost, and inadequate health-seeking behaviors.

## 5 Conclusions

Most patients with cervical cancer were diagnosed at a late stage owing to the lack of cervical cancer awareness, including knowledge about early symptoms, lack of routine screening, and delayed treatment owing to social barriers. Healthcare systems should undertake initiatives to educate patients and improve access to healthcare. We hope the government can employ stricter policies to implement cervical cancer detection and prevention. We support the government policy to vaccinate elementary students with HPV vaccine and increase coverage of the pap test.

## Funding

The authors declare no funding for this research.

## CRediT authorship contribution statement

**Brahmana Askandar Tjokroprawiro:** Conceptualization, Writing – review & editing, Supervision. **Khoirunnisa Novitasari:** Formal analysis, Validation, Writing – original draft. **Wita Saraswati:** . **Indra Yuliati:** Conceptualization, Supervision. **Renata Alya Ulhaq:** Project administration, Writing – review & editing. **Hanif Ardiansyah Sulistya:** Visualization.

## Declaration of competing interest

The authors declare that they have no known competing financial interests or personal relationships that could have appeared to influence the work reported in this paper.
